# During acute experimental infection with the reticulotropic *Trypanosoma cruzi* strain Tulahuen IL-22 is induced IL-23-dependently but is dispensable for protection

**DOI:** 10.1038/srep32927

**Published:** 2016-09-21

**Authors:** Hanna Erdmann, Jochen Behrends, Christoph Hölscher

**Affiliations:** 1 Division of Infection Immunology, Research Centre Borstel, Borstel, Germany; 2 Priority Area Infection, Research Centre Borstel, Borstel, Germany; 3 Cluster of Excellence Inflammation-at-Interfaces (Borstel-Kiel-Lübeck-Plön), Germany; 4 Fluorescence Cytometry Core Facility, Research Centre Borstel, Borstel, Germany

## Abstract

Protective immunity against *Trypanosoma cruzi*, the causative agent of Chagas disease, depends on the activation of macrophages by IFN-γ and IL-17A. In contrast, IL-10 prevents immunopathology. IL-22 belongs to the IL-10 cytokine family and has pleiotropic effects during host defense and immunopathology, however its role in protection and pathology during *T. cruzi* infection has not been analyzed yet. Therefore, we examined the role of IL-22 in experimental Chagas disease using the reticulotropic Tulahuen strain of *T. cruzi*. During infection, IL-22 is secreted by CD4-positive cells in an IL-23-dependent fashion. Infected IL-22^−/−^ mice exhibited an increased production of IFN-γ and TNF and displayed enhanced numbers of activated IFN-γ-producing T cells in their spleens. Additionally, the production of IL-10 was increased in IL-22^−/−^ mice upon infection. Macrophage activation and by association the parasitemia was not affected in the absence of IL-22. Apart from a transient increase in the body weight loss, infected IL-22^−/−^ mice did not show any signs for an altered immunopathology during the first fourteen days of infection. Taken together, although IL-22 is expressed, it seems to play a minor role in protection and pathology during the acute systemic infection with the reticulotropic Tulahuen strain of *T. cruzi*.

The obligatory intracellular parasite *Trypanosoma cruzi* is the causative agent of Chagas disease, which is common in Central and South America. Protective immune responses against *T. cruzi* are based on the release of interleukin (IL)-12 and IL-23 by *T. cruzi*-infected macrophages^1^,^2^,^3^. IL-12 induces the interferon-gamma (IFN-γ)-production from natural killer (NK) and T cells^4^. IFN-γ activates nitric-oxide-synthase (NOS)2 expression and the production of reactive nitrogen intermediates (RNI) in macrophages, which is critical for their trypanocidal activity^5^,^6^. Accordingly, IFN-γ receptor (R)-deficient (^−/−^) and NOS2^−/−^ mice exhibit an increased susceptibility during *T. cruzi* infection^7^. Another IL-12-induced cytokine is tumor-necrosis-factor (TNF) that plays a role in amplifying RNI-production of IFN-γ-activated macrophages and thus contributes to protection^8^. IL-6 is also produced during experimental *T. cruzi* infection and contributes to protection^9^, possibly by activating endothelial cells to up-regulate adhesion molecules for lymphocyte migration^10^. Among other chemokines, the chemokines CXC chemokine ligand 9 (CXCL9) and CXCL10 promote protective immune responses against *T. cruzi* as the neutralization of these chemokines resulted in an increased parasitemia^11^. Induced by IL-23, IL-17A also acts on macrophages and stimulates their phagocytic activity leading to an enhanced killing of intracellular *T. cruzi* parasites^12^. Consistently, IL-17A^−/−^ mice are highly susceptible to *T. cruzi* infection showing an increased parasitemia as well as an increased mortality rate upon infection^12^,^13^. IL-17A-secretion *in vivo* was shown to be IL-23-dependent as IL-23p19^−/−^ mice produced significantly less IL-17A compared to C57BL/6 wild-type mice^12^. The control of *T. cruzi* replication is remarkably compromised in IL-23p19^−/−^ mice and this effect was ascribed to the reduced IL-17A production^12^. However, since the production of IL-22 is also dependent on IL-23^14^,^15^, this cytokine might also contribute to protection or pathology during experimental *T. cruzi* infection.

IL-22 belongs to the IL-10 family of cytokines, which also comprises IL-10, IL-19, IL-20, IL-24 and IL-26^16^. Its expression is restricted to a subset of activated immune cells, including T cells, especially T helper (Th)17 cells, and NK cells^14^,^17^,^18^. IL-22 signals through a heterodimeric receptor consisting of IL-10Rβ and IL-22R. Whereas IL-10Rβ is ubiquitously expressed, IL-22R expression seems to be restricted to epithelial cells, keratinocytes and fibroblasts^19^. Consequently, IL-22 plays a major role in the host defense against pathogens at barrier surfaces, where it supports barrier integrity, regulates wound repair and stimulates the production of antimicrobial peptides^20^,^21^,^22^,^23^. For example, IL-22 protects the mucosal airway surface against infection with the Gram-negative bacterium *Klebsiella pneumodiae*^23^. IL-22 can also reduce tissue destruction in a noninfectious model of conA-induced hepatitis, where it protects the liver by enhancing the growth and survival of hepatocytes^24^. However, in contrast to its protective activity, IL-22 can also promote pathological inflammation, like promoting dermal inflammation in a T-cell transfer-model of psoriasis-like inflammation^25^. Regarding to parasitic infections, it was reported that IL-22 contributes to pathogenic inflammation in the intestine during oral *Toxoplasma gondii*-infection^26^.

With respect to the pleiotropic roles of IL-22 in host defense, tissue repair and pathological inflammation, we were interested in the role of IL-22 in acute experimental *T. cruzi* infection. *T. cruzi* infects different tissues and causes pathology in various organs such as the heart, the gastrointestinal tract, the spleen and the liver. The parasite’s genetic variability decides on tissue tropism and the outcome of disease. Reticulotropic strains prefer to invade phagocytic mononuclear cells (e.g. of the spleen and the liver) whereas myotropic strains tend to invade muscle cells (e.g. of the heart). Since IL-22 is known to be involved in protection of hepatocytes during hepatitis^24^, we here used the reticulotropic *T. cruzi* strain Tulahuen that mainly infects spleen and liver cells and induces splenomegaly and liver pathology^27^,^28^,^29^,^30^,^31^ to analyze the role of this cytokine for protection and/or pathology during experimental Chagas disease.

## Results

### IL-23-dependent production of IL-22 during experimental *T. cruzi* infection

**Figure 1:**
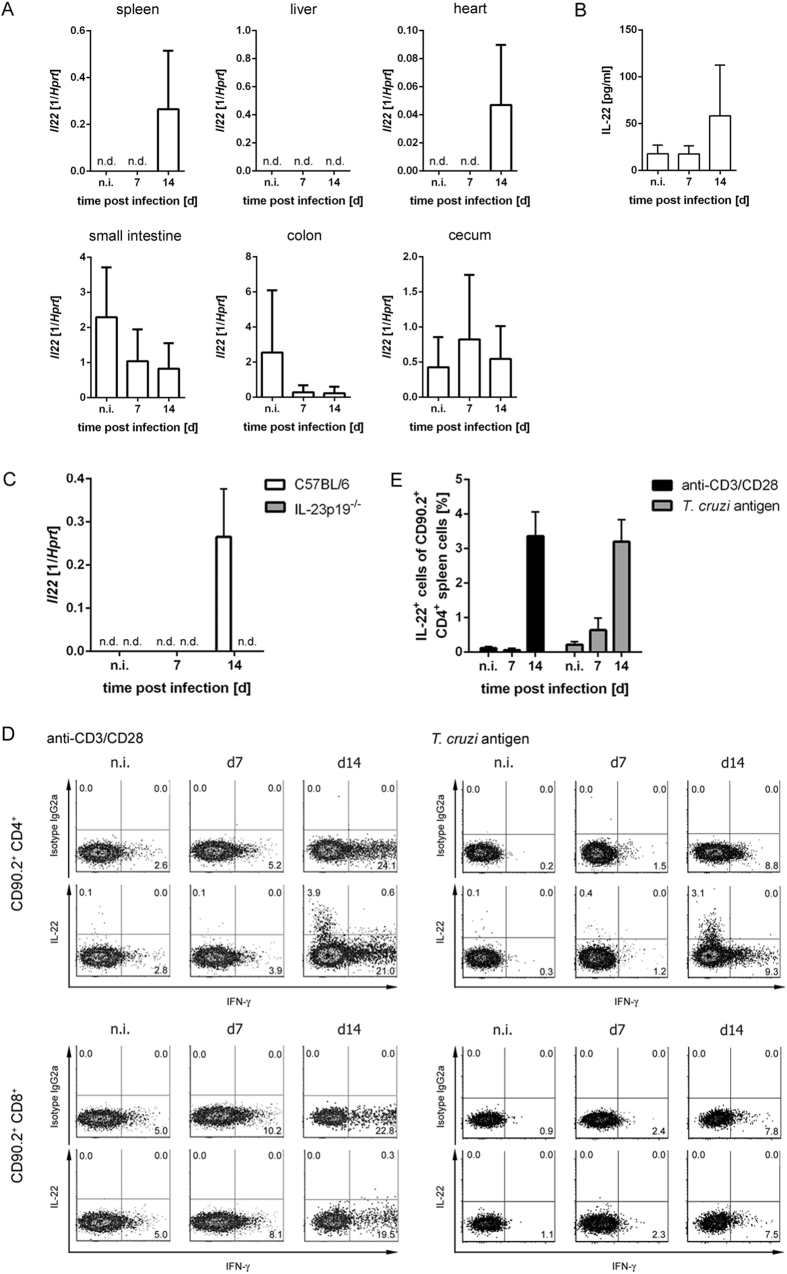
IL-22 is produced in response to *T. cruzi* infection. C57BL/6 mice were infected i.p. with 500 *T. cruzi* trypomastigotes. (**A**) *Il22* mRNA expression in the spleen, liver, heart, small intestine, colon and cecum was quantified by real-time PCR. (**B**) The concentration of IL-22 in the sera was determined by cytometric bead array. (**C**) C57BL/6 mice and IL-23p19^−/−^ mice were infected i.p. with 500 *T. cruzi* trypomastigotes and the *Il22* mRNA expression in spleens was quantified by real-time PCR at the indicated time points post infection. Results are expressed as means ± standard deviations of 4–5 mice per group. (**D**) The intracellular IL-22 and IFN-γ production of C57BL/6 spleen cells was analyzed by flow cytometry after anti-CD3/CD28 stimulation or specific stimulation with *T. cruzi* antigen. Cells were gated on CD90.2^+^ CD4^+^ or CD90.2^+^ CD8^+^ T cells, respectively. Shown are representative dot plots of from 5 mice per group. (**E**) Percentages of IL-22-producing cells out of CD90.2^+^ CD4^+^ T cells after anti-CD3/CD28 stimulation or after antigen-specific stimulation (as shown in (**D**)). Results are expressed as means ± standard deviations of 5 mice per group. n.d. not detectable; n.i. not infected.

We first determined whether IL-22 was induced during experimental *T. cruzi* infection and analyzed the *Il22* gene expression in spleen, liver, heart, small intestine, colon and cecum of naïve C57BL/6 mice and mice infected with 500 *T. cruzi* trypomastigotes (Fig. 1A). *Il22* mRNA was induced in the spleen and in the heart on day 14 post infection, whereas it was not detectable in the liver during the infection (Fig. 1A). In contrast, *Il22* mRNA was already expressed in intestinal structures in naïve mice and seemed to be downregulated in response to *T. cruzi* infection in the small intestine and the colon (Fig. 1A). Additionally, we observed increased levels of IL-22 in the sera of C57BL/6 mice after 14 days of infection (Fig. 1B). We next determined whether the production depends on IL-23. Therefore, we infected C57BL/6 and IL-23p19^−/−^ mice i.p. with 500 *T. cruzi* trypomastigotes and analyzed *Il22* gene expression in spleens of naïve and infected mice. *Il22* mRNA was expressed in spleens of C57BL/6, but not of IL-23p19^−/−^ mice, 14 days after the infection, indicating that IL-22 is produced during *T. cruzi* infection in an IL-23-dependend manner (Fig. 1C). To further characterize IL-22-producing cells, spleen cells of naïve and infected C57BL/6 mice were stimulated with anti-CD3/CD28 or with *T. cruzi* antigen and were analyzed by flow cytometry. After exclusion of doublets (CD90.2 vs. SSC-W and FSC-A vs. FSC-H) and autofluorescent cells, cells were gated on CD90.2^+^ CD4^+^ or CD90.2^+^ CD8^+^ cells, respectively, and were analyzed for IL-22- and IFN-γ-production (Fig. 1D). Whereas both CD4^+^ and CD8^+^ T cells produced IFN-γ during *T. cruzi* infection, only CD4^+^ T cells were positive for IL-22 at day 14 post infection after unspecific or antigen-specific stimulation (Fig. 1D,E).

### Increased T cell activation and enhanced secretion of pro-and anti-inflammatory cytokines in *T. cruzi*-infected IL-22^−/−^ mice

**Figure 2:**
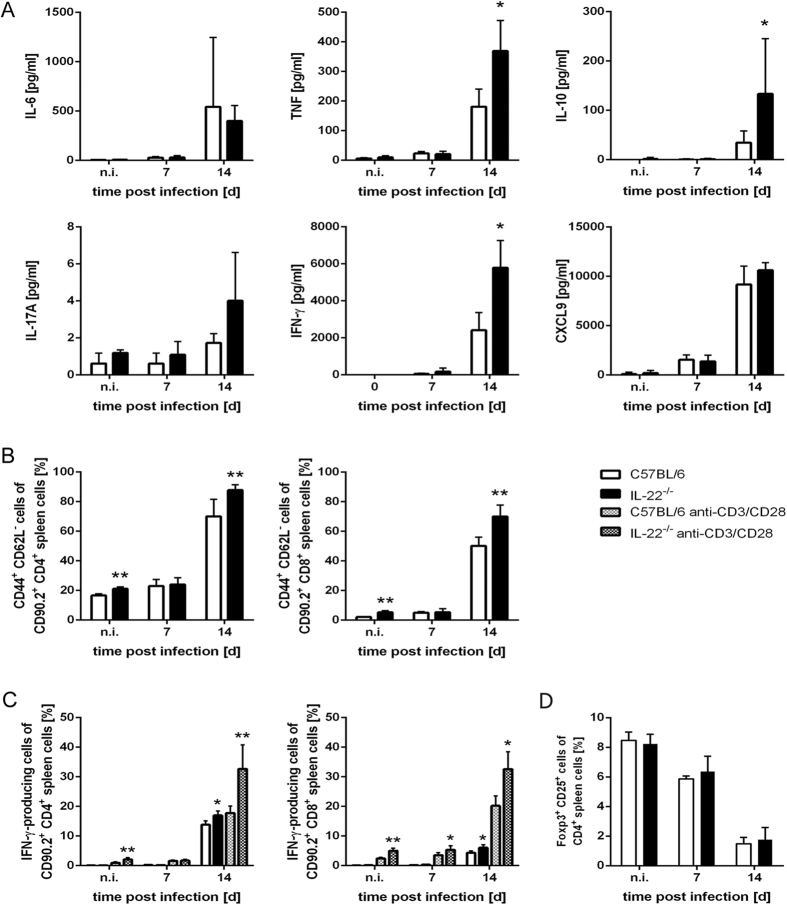
Increased cytokine and chemokine production as well as enhanced numbers of activated T cells in IL-22^−/−^ mice during *T. cruzi* infection. C57BL/6 mice and IL-22^−/−^ mice were infected i.p. with 500 *T. cruzi* trypomastigotes. (**A**) Single cell suspensions of spleen cells were cultured for 24 h and the secretion of different cytokines and chemokines into the supernatant was determined by cytometric bead array. (**B**) Spleen cells were analyzed by flow cytometry at the indicated time points after infection. Shown is the percentage of activated (CD44^+^ CD62L^−^) cells of CD90.2^+^ CD4^+^ or CD90.2^+^ CD8^+^ spleen cells, respectively. (**C**) Spleen cells were stimulated with anti-CD3/CD28 or left unstimulated and were analyzed for intracellular IFN-γ production. Shown is the percentage of IFN-γ-producing cells of CD90.2^+^ CD4^+^ or CD90.2^+^ CD8^+^ spleen cells, respectively. (**D**) The percentage of Foxp3^+^ CD25^+^ T_regs_ out of CD4^+^ T cells was analyzed by flow cytometry. Results are expressed as means ± standard deviations of 5 mice per group. * and ** indicate statistical significance (p ≤ 0.05 and p ≤ 0.01, respectively) compared to C57BL/6 wild-type mice.

We next assessed whether IL-22 deficiency influenced cytokine and chemokine production during *T. cruzi* infection (Fig. 2). Whereas the secretion of IL-6 by spleen cells was unaffected by the lack of IL-22, the splenic production of IFN-γ, TNF and IL-10 was increased in IL-22^−/−^ mice compared to wild-type mice at day 14 post infection. IL-17A was almost undetectable in both mouse groups during the infection (Fig. 2A). As recombinant IL-22 was previously shown to decrease *Cxcl9* expression during lung fibrosis^32^, we also analyzed the CXCL9 secretion by spleen cells of infected C57BL/6 and IL-22^−/−^ mice. However, we found an unaltered CXCL9 chemokine production upon IL-22 deficiency (Fig. 2A). To determine, whether the IL-22 deficiency, influences T cell recruitment during *T. cruzi* infection, we examined splenic T cell populations by flow cytometry. In comparison to C57BL/6 animals, IL-22^−/−^ mice displayed enhanced percentages of activated (CD44^+^ CD62L^−^) CD4^+^ and CD8^+^ T cells in the spleens (Fig. 2B). Additionally, flow cytometric analyses revealed enhanced percentages of IFN-γ-secreting splenic CD4^+^ and CD8^+^ T cells in *T. cruzi*-infected IL-22^−/−^ mice over C57BL/6 mice (Fig. 2C). We also analyzed whether the percentages of regulatory T cells (T_regs_) were affected by the absence of IL-22. Whereas effector T cells expand during *T. cruzi* infection in C57BL/6 mice, Foxp3^+^ T_regs_ exhibit a decreased proliferation rate and the ratio between T_regs_ and effector T cells decreases during the infection^33^. This effect was also observed in C57BL/6 and in IL-22^−/−^ mice, where the percentages of Foxp3^+^ CD25^+^ T cells out of total CD4^+^ spleen cells decreased over the course of the infection (Fig. 2D). However, there was no difference in the percentage of T_regs_ between C57BL/6 and IL-22^−/−^ mice (Fig. 2D), suggesting that IL-22 does not affect the development of T_regs_ in non-infected mice and during *T. cruzi* infection. Taken together, we observed an increased secretion of TNF, IFN-γ, IL-10 as wells as an enhanced number of IFN-γ-producing T cells in the spleens of infected IL-22^−/−^ mice.

### IL-22 does not influence macrophage activation during *T. cruzi* infection

**Figure 3:**
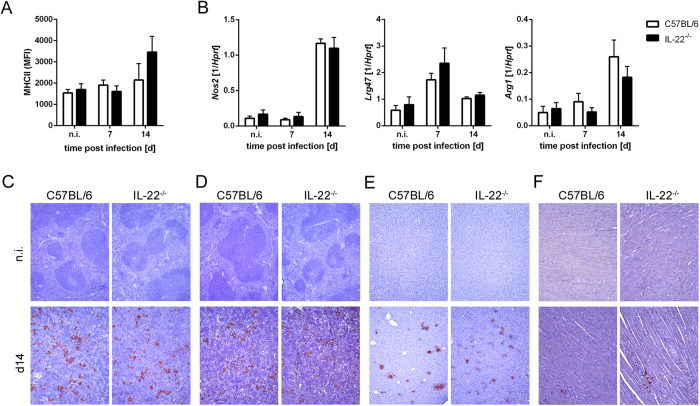
Unaltered macrophage activation in *T. cruzi*-infected IL-22^−/−^ mice. C57BL/6 mice and IL-22^−/−^ mice were infected i.p. with 500 *T. cruzi* trypomastigotes. (**A**) The mean fluorescent intensity (MFI) of MHCII (I-A/I-E) expression on splenic macrophages (CD90.2^−^, CD11b^+^, CD11c^+^, Gr1^−^, MHCII^+^) was detected by flow cytometry. (**B**) mRNA expression of *Nos2*, *Lrg47* and *Arg1* in spleens was analyzed by real-time PCR at the indicated time points post infection. Similar results were obtained in two independent experiments. Results are expressed as means ± standard deviations of 5 mice per group (2–5 uninfected mice per group). (**C**) Organ sections of spleen (**C**,**D**), liver (**E**) and heart (**F**) were stained for NOS2 (**C**,**E**,**F**) and for ARG1 (**D**) and counterstained with hematoxylin. Representative sections from five mice per group (2 uninfected mice per group) are shown. Magnification: spleen, liver 100x; heart 200x.

Macrophages represent an important site for *T. cruzi* replication in the acute phase of infection, especially for reticulotropic *T. cruzi* strains such as the Tulahuen strain. However, IFN-γ-activated macrophages are the main effector cells that control *T. cruzi* replication by the production of RNI, which kill intracellular parasites by chemically modifying the structural properties of *T. cruzi* macromolecules. Additionally, the IFN-γ-inducible p47GTPase LRG-47 is directly involved in killing of parasites by macrophages^34^. Because we found increased numbers of IFN-γ-producing cells in the spleens of IL-22^−/−^ mice, we next analyzed the macrophage activation of *T. cruzi*-infected C57BL/6 and IL-22^−/−^ mice. Flow cytometric analysis revealed that the numbers of macrophages (CD90.2^−^, CD11b^+^, CD11c^+^, Gr1^−^, MHCII^+^) were unaffected upon IL-22 deficiency (C57BL/6 mice, 0.98 × 10^6^ ± 0.47 × 10^6^; IL-22^−/−^ mice, 1.14 × 10^6^ ± 0.46 × 10^6^ on day 14 post infection). Furthermore, macrophages from IL-22^−/−^ mice exhibited an unaltered MHCII expression in comparison to wild-type macrophages during infection (Fig. 3A). Additionally, the gene expression of *Nos2* and *Lrg47*, encoding the main proteins with trypanocidal activities NOS2 and LRG-47, did not differ between wild-type and IL-22^−/−^ mice (Fig. 3B). Gene expression of *Arg1* encoding ARG1, which promotes intracellular growth of *T. cruzi* parasites^35^,^36^ was also identical in infected IL-22^−/−^ and C57BL/6 mice (Fig. 3B). Accordingly, histological analysis revealed unchanged NOS2 expression in spleens, livers and hearts (Fig. 3C,E,F) as well as a similar ARG1 expression in spleens (Fig. 3D) in both mouse groups, whereas ARG1 was not expressed in heart sections (data not shown). Thus, IL-22 deficiency does not alter MHC-II, NOS2 and ARG1 expression during *T. cruzi* infection *in vivo*.

### IL-22 has no effect on parasite killing by *in vitro*-infected macrophages

**Figure 4:**
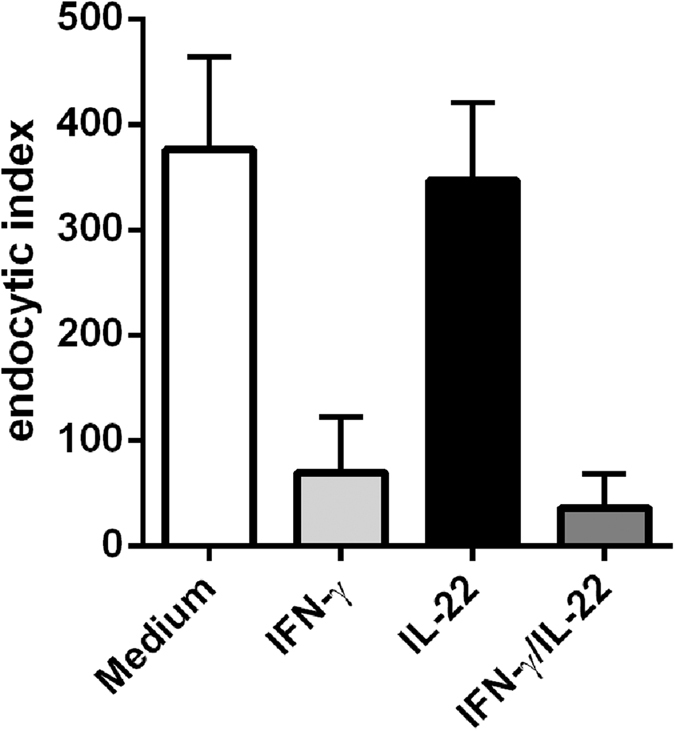
Recombinant IL-22 does not influence parasite killing by macrophages *in vitro*. BMDMs were treated with recombinant IL-22, IFN-γ or both cytokines for 2 h and were subsequently infected with *T. cruzi* culture trypomastigotes. Extracellular parasites were removed at 2 h post infection and the cells were again incubated in the presence of the respective cytokines. 48 h post infection, the endocytic index (percentage of infected cells multiplied by the average number of intracellular amastigotes) was determined after Hemacolor staining. Results are expressed as means ± standard deviations of triplicate cultures.

As we have published earlier, IL-17A stimulates parasite killing in macrophages without inducing *Nos2* or *Lrg47* expression^12^. It rather stimulates the phagocytic activity of macrophages. Thus, we next assessed whether IL-22 contributes to parasite killing by influencing the intracellular fate of *T. cruzi* in macrophages. In line with this, IL-22 was shown to enhance the phagolysosomal fusion in *Mycobacterium tuberculosis*-infected human macrophages and inhibit mycobacterial growth^37^. To test the direct effect of IL-22 on macrophages, we stimulated BMDMs with recombinant IL-22, IFN-γ as a control or both cytokines for 2 h and infected them with *T. cruzi* culture trypomastigotes (Fig. 4). Extracellular parasites were removed at 2 h post infection and the cells were again incubated in the presence of the respective cytokines. At 48 h post infection we determined the endocytic index, which corresponds to the percentage of infected cells multiplied by the average number of intracellular amastigotes. Unstimulated BMDMs exhibited a high endocytic index, suggesting that the parasites survived and replicated within these cells. In contrast, IFN-γ-stimulated BMDMs were obviously able to kill parasites, as the endocytic index was clearly reduced. However, IL-22 did neither influence the endocytic index of unstimulated nor of IFN- γ-stimulated macrophages. Therefore we concluded, that IL-22 does not directly enhance parasite killing in unstimulated or stimulated macrophages.

### Control of *T. cruzi* infection is not affected by the absence of IL-22

**Figure 5:**
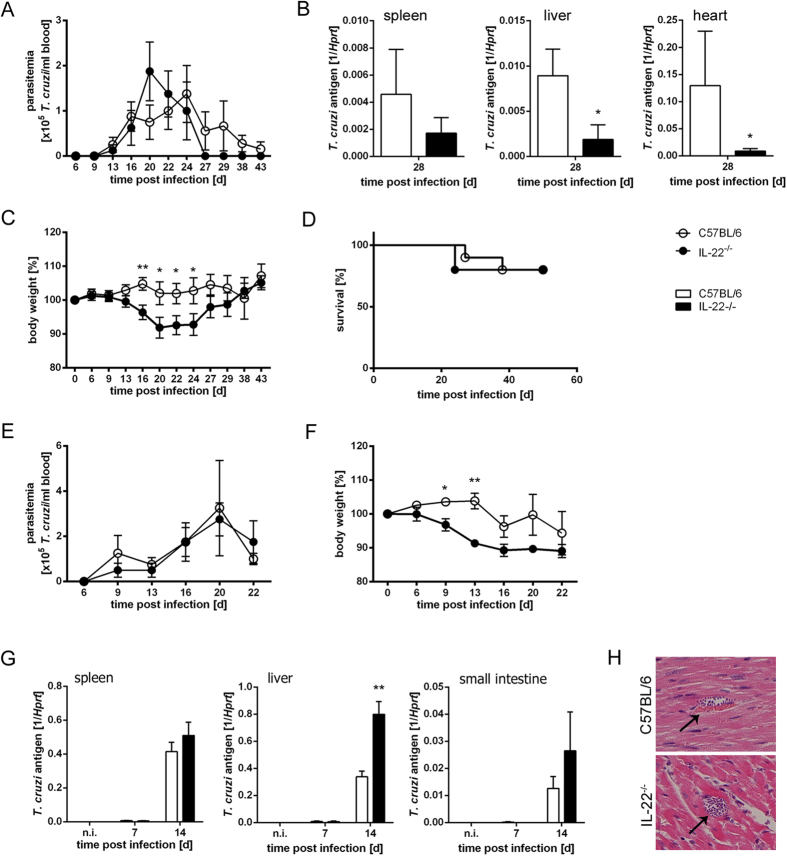
Undiminished control of *T. cruzi* infection. In (**A–D**) C57BL/6 and IL-22^−/−^ mice were infected i.p. with a sublethal dose of 50 *T. cruzi* blood trypomastigotes. Parasitemia (**A**), tissue parasite burdens (**B**) body weight changes (**C**) and survival (**D**) were assessed during the acute infection. Results are expressed as means ± standard deviations of 10 mice per group. One out of three independent experiments with comparable results is shown. In (**E–H**) C57BL/6 and IL-22^−/−^ mice were infected i.p. with a high dose of 500 *T. cruzi* blood trypomastigotes. Parasitemia (**E**), body weight changes (**F**) and tissue parasite burdens (**G**) were assessed during the acute infection. Results are expressed as means ± standard deviations of 5 mice per group (2 uninfected mice per group). (**H**) H&E-stained heart sections were analyzed for the presence of parasite nests (arrows). 5 mice per group were analyzed. Magnification 400x. * and ** indicate statistical significance (p ≤ 0.05 and p ≤ 0.01, respectively) compared to C57BL/6 wild-type mice.

We next assessed the outcome of acute *T. cruzi* infection in C57BL/6 and IL-22^−/−^ mice. To analyze the parasitemia, tissue burdens, mortality and body weight changes, we injected either 50 parasites i.p., which corresponds to a dose below the 50% lethal dose of 75 parasites (Fig. 5A–D) or a higher dose of 500 blood-trypomastigotes, respectively (Fig. 5E–H). The parasitemia did not differ between C57BL/6 and IL-22^−/−^ mice during the course of low dose infection (Fig. 5A). However, IL-22^−/−^ mice seemed to be more efficient in clearing the parasites as no trypomastigotes were detectable at day 27 post infection in IL-22^−/−^ mice, whereas C57BL/6 mice exhibited detectable numbers of parasites until day 43 post infection. Accordingly, parasite burdens in spleens, livers and hearts were decreased at day 28 post infection in the absence of IL-22 (Fig. 5B). IL-22^−/−^ mice showed a significantly increased body weight loss during early infection (Fig. 5C), which could be a sign for pathology. However the increased body weight loss in infected IL-22^−/−^ mice was only transient and did not influence the mortality rate (Fig. 5D). The same effects were also seen during an infection with 500 parasites. IL-22^−/−^ mice were able to control *T. cruzi* infection (Fig. 5E), however they also exhibited an increase in body weight loss (Fig. 5F). The analysis of tissue burdens during early infection with 500 parasites revealed an unaltered parasite burden in the spleens and the small intestines and increased burdens in the livers of IL-22^−/−^ mice (Fig. 5G). This is in accordance with the parasitemia, which seems to be unaltered or even slightly increased in IL-22^−/−^ mice during early infection. Microscopic analysis of hematoxylin/eosin (H&E)-stained heart sections revealed that detectable parasite nests were very rare (only present in one of five mice in both groups) and similar in both mouse groups (Fig. 5H).

As NOS2, LRG-47 and ARG1 expression by macrophages was unaltered *in vivo* in IL-22^−/−^ mice and IL-22 did not influence parasite killing by macrophages *in vitro*, the fact that the parasitemia was unaffected by the absence of IL-22 was not surprising. However, the enhanced body weight loss in IL-22^−/−^ mice indicated a role of this cytokine in regulating the development of immunopathology during the acute infection.

### Unaltered immunopathology of IL-22^−/−^ mice during acute *T. cruzi* infection

**Figure 6:**
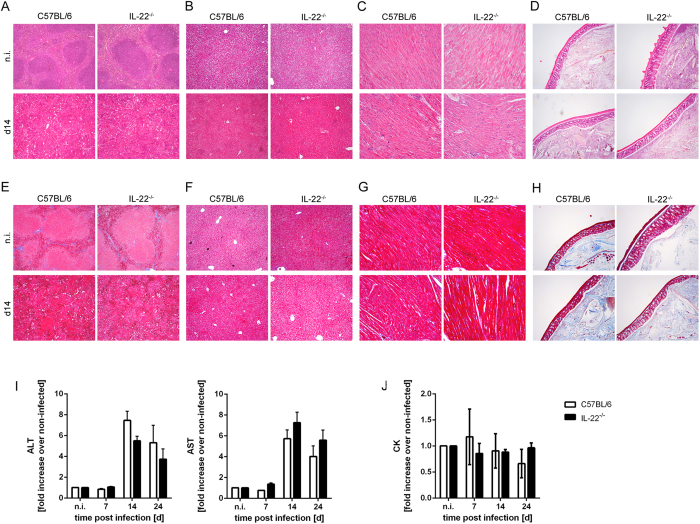
Unaltered pathology in *T. cruzi*-infected IL-22^−/−^ mice. C57BL/6 mice and IL-22^−/−^ mice were infected i.p. with 500 *T. cruzi* trypomastigotes. Tissue sections of spleen (**A**,**E**), liver (**B**,**F**), heart (**C**,**G**) and colon (**D**,**H**) were stained with H&E (**A**–**D**) or with azan trichrome (**E**–**H**). Representative sections from five mice per group (2 uninfected mice per group) are shown. Magnification: spleen, liver, colon 100x; heart 200x. Liver derived enzymes ALT and AST (**I**) and CK (**J**) were quantified in sera at the indicated time points after infection. Results are expressed as means ± standard deviations of 5 mice per group (2–5 uninfected mice per group). Similar results were obtained in two independent experiments.

*T. cruzi* infects multiple organs during the acute infection and causes organ pathology. Particularly the reticulotropic Tulahuen strain induces liver pathology and splenomegaly during the acute phase of experimental Chagas disease^27^,^28^,^29^,^30^,^31^. Since IL-22 is known to be involved in tissue repair, wound healing and protection from hepatitis^24^, we hypothesized that the *T. cruzi* Tulahuen-induced pathology may be altered in the absence of IL-22. To address this issue, we analyzed spleen, liver and heart sections microscopically after high dose infection with 500 *T. cruzi* blood trypomastigotes (Fig. 6A–C). We observed a destruction of the splenic architecture after 14 days of infection, which was comparable in both mouse groups (Fig. 6A). Inflammatory cell infiltration into the liver and the heart observed on day 14 post infection was similar in C57BL/6 and IL-22^−/−^ mice (Fig. 6B,C). Since *T. cruzi* parasites were also found in intestinal structures (Fig. 5G), we also analyzed H&E-stained sections of the colon (Fig. 6D), the small intestine and the cecum (data not shown) for pathological changes, e.g. dead cells in the lumen, changes of the surface epithelium (ulceration, desquamation), of the mucosa (inflammatory infiltration) and the submucosa (edema). However, the colon (Fig. 6D) as well as the small intestine or cecum (data not shown), did not show any signs of pathology. To further assess pathological alterations, we analyzed collagen deposition in the different organs. An azan trichrome stain revealed only poor collagen deposition in spleen, liver, heart and colon, which was not influenced by the lack of IL-22 (Fig. 6E–H). Consistent with our microscopic analysis, serum levels of the aspartate transaminase (AST) and the alanine transaminase (ALT) (indicating liver destruction) increased during the course of infection with a peak at day 14 post infection, but were similar in both mouse groups (Fig. 6I). In accordance with the reticulotropic characteristic of the Tulahuen strain, we did not observe an induction of serum creatine kinase (CK) levels in both mouse groups during the first 24 days post infection, which would be an indicator of heart and skeletal muscle damage^38^,^39^. Taken together, IL-22 has no significant impact on the *T. cruzi*-induced acute immunopathology.

## Discussion

The anti-inflammatory cytokine IL-10 plays a major role during experimental *T. cruzi* infection by regulating overshooting pro-inflammatory immune responses and preventing immunopathology^29^,^40^. However, the role of the closely related cytokine IL-22 during *T. cruzi* infection was not investigated before. IL-22 is a pleiotropic cytokine that on the one hand supports host resistance and tissue repair, but on the other hand is also involved in pathological inflammation^20^,^21^,^22^,^23^,^24^,^25^,^26^. We here demonstrate that IL-22 is produced during *T. cruzi* infection and analyzed the outcome of acute systemic *T. cruzi* infection in IL-22^−/−^ mice using the reticulotropic Tulahuen strain of *T. cruzi*.

IL-22 deficiency was accompanied by an increased production of the pro-inflammatory cytokines TNF and IFN-γ during *T. cruzi* infection. The chemokine CXCL9 is important for the migration of Th1 cells and has been shown to promote protective immune responses during experimental *T. cruzi* infection^11^. A previous study showed that recombinant IL-22 is able to decrease *Cxcl9* expression^32^, maybe by acting on epithelial cells that bear the IL-22 receptor. In that study, IL-22 protects against lung fibrosis by suppressing the CXCL9-dependent recruitment of CD4^+^ T cells into the lung. During *T. cruzi* infection, IL-22 also controls recruitment of T cells into spleens as we observed increased numbers of activated T cells, as well as enhanced percentages of IFN-γ-secreting T cells in the spleens upon IL-22 deficiency. However, this seems to be independent on CXCL9, as we found an unaltered CXCL9 secretion by the lack of IL-22 during infection.

Nevertheless, not only the secretion of pro-inflammatory cytokines was enhanced, but also the production of the anti-inflammatory cytokine IL-10 was increased in IL-22^−/−^ mice during *T. cruzi* infection. IL-10 can directly act on macrophages and inhibit the IFN-γ-induced expression of NOS2, thereby inhibiting parasite killing^41^. This might be the reason that we observed an undiminished NOS2 and LRG-47 expression in infected IL-22^−/−^ mice despite increased production of IFN-γ.

Additionally, we did not observe direct effects of recombinant IL-22 on parasite killing by macrophages *in vitro*, as we have previously shown for IL-17A^12^. Accordingly, our data reveal that IL-22 is dispensable for control of *T. cruzi* growth *in vivo*.

*T. cruzi* infects several different organs and causes organ pathology. Whereas myotropic *T. cruzi* strains preferably infect cardiomyocytes and cause heart pathology, reticulotropic *T. cruzi* strains, such as the Tulahuen strain we used, are more found in the spleen and liver, where they can induce severe hepatic pathology and splenomegaly. IL-22 is directly involved in tissue remodeling and for example protects hepatocytes during conA-induced hepatitis^42^. Furthermore, like IL-10, IL-22 might also regulate overshooting pro-inflammatory immune responses and hereby protects from pathology. As we observed a temporary increased weight loss during the infection, we hypothesized that the acute immunopathology might be altered in the absence of IL-22.

In accordance with the reticulotropic character of the Tulahuen strain, we observed a destruction of the splenic architecture and inflammatory cell infiltration into the liver as well as increased AST and ALT levels in both mouse groups during the course of infection. However, we did not find any differences between infected C57BL/6 and IL-22^−/−^ mice. In accordance with the finding that the increased body weight loss is only transient and the mice recovered, the AST and ALT levels begin to decrease again at day 24 post infection. As we did not observe *Il22* mRNA expression in the liver, IL-22 is obviously not produced in response to *T. cruzi* infection to protect hepatocytes from *T. cruzi*-induced hepatic pathology. However, as we observed an induction of *Il22* mRNA expression in the heart during infection, we also analyzed acute heart pathology. In contrast to myotropic *T. cruzi* strains, which induce an extensive damage to the myocardium and an increase of CK serum levels^38^, the Tulahuen strain we used here failed to induce histopathological changes in the heart and an increase in CK levels during the first 24 days of infection. As the body weight loss was only transient and the mice recovered, we assume that mice will not develop severe heart pathology at later time points. As IL-22 is involved in intestinal inflammation at day 8 after *T. gondii* infection^26^,^43^, we also analyzed intestinal structures in *T. cruzi*-infected mice. Although *T. cruzi* parasites were present in intestinal structures as shown by real-time PCR (Fig. 5G), these structures did not show any signs of pathology in both mouse groups (Fig. 6D,H).

Taken together, we did not find any differences in pathology between IL-22^−/−^ and wild-type mice. However, the lack of IL-22 might be compensated by an increased IL-10 production, a cytokine that is known to ameliorate immunopathology during *T. cruzi* infection^29^,^40^. The reason, why IL-22^−/−^ mice exhibited an increased body weight loss during infection, but not an increased pathology in compensation to wild-type mice is unclear. However, since the increased body weight loss is only temporary and the IL-22^−/−^ mice recovered, differences in the pathology might be so sparse that it is not detectable with our methods. A pathology, which is detectable by histology, would presumably not lead to a recovery and a survival of mice.

In accordance with an unaltered pathology, survival rates were not influenced by the absence of IL-22 after infection with 50 parasites. Due to the fact, that the parasitemia and the pathology was also unaltered between both mouse groups after infection with 500 parasites, we would also expect an unaltered mortality in mice infected with this higher dose.

IL-22 rather mediates its protective effects at barrier surfaces and seems to play a minor role during systemic infections. Consistently, it has been reported, that IL-22^−/−^ mice are resistant to systemic *T. gondii*-infection, but are susceptible after oral infection with this parasite^26^. In this respect, although IL-22 seemed to be dispensable for the development of immunity against systemic *T. cruzi* infection, it might play a role when mice are infected by other routes. The ingestion of parasite-contaminated food via the oral route is an important mode of transmission to humans in some areas^44^. Therefore, it will be interesting to analyze protection and pathology of IL-22^−/−^ mice during oral *T. cruzi* infection in the future. In terms of pathology, IL-22 mediates protection in the gastrointestinal tract in inflammatory bowel disease^22^, but induces harmful immune responses during oral *T. gondii* infection leading to pathology^26^. Thus, during *T. cruzi* infection IL-22 may induce either harmful or helpful inflammatory responses. In terms of protection, it is conceivable that IL-22-induced production of antimicrobial peptides in intestinal structures might protect from oral *T. cruzi* infection. Although it is not clear whether *T. cruzi* is susceptible to mouse antimicrobial peptides, it has clearly been demonstrated that certain mammalian antimicrobial peptides, like the porcine antimicrobial peptide NK-lysin^45^ and the human defensin α-1^46^, can cause *T. cruzi* destruction by disruption of the surface membrane integrity.

Furthermore, our study did not include experiments to determine the role of IL-22 during chronic *T. cruzi* infection. Anti-inflammatory cytokines, especially IL-10, are known to mediate protective effects during the chronic phase of disease, for example it was demonstrated that patients, who remain asymptomatic, display an increased IL-10 expression in comparison to patients with cardiac disease and are capable of downmodulating immune responses in a way that limits pathology^47^. Thus, it will be interesting to analyze the role of IL-22, a member of the IL-10 family, in a mouse model of chronic heart disease using myotropic *T. cruzi* strains in the future.

Taken together, although IL-22 is expressed during *T. cruzi* infection, it plays a minor role in protection and pathology during the acute phase of experimental infection with the Tulahuen strain.

## Methods

### Mice and Parasites

IL-23p19^−/− ^48 and IL-22^−/− ^49 breeding pairs were kindly provided by Nico Ghilardi (Genentech, South San Francisco, CA USA) and Jean-Christophe Renauld (Ludwig Institute for Cancer Research, Brussels, Belgium), respectively. Both strains were on a C57BL/6 background. Mutant mice and C57BL/6 wild-type mice were bred under specific-pathogen-free conditions in the animal facility of the Research Centre Borstel. For infection experiments the *T. cruzi* strain Tulahuen (WHO reference strain M/HOM/CH/00/Tulahuen C2) was used. To avoid genetic modifications of the parasite strain by serial passages in mice a master stock of blood trypomastigotes obtained from infected immunodeficient animals was stored. This approach allowed us to preserve a stable virulence of the strain still with a LD50 of 75 parasites. To obtain high numbers of *T. cruzi* blood form trypomastigotes for infection experiments and to prevent the transfer of lymphocytes and antibodies, SCID mice (purchased from Charles River, Sulzfeld, Germany) were infected i.p. with aliquots of these cryopreserved *T. cruzi* stabilates. On day 12 post infection, blood was collected from infected SCID mice, mixed with heparin, and parasites were enriched in the plasma by differential centrifugation. Parasites were resuspended in PBS/0.5% Glucose and used for i.p. infection of female mice aged 8 to 12 weeks. An infection dose of 500 blood trypomastigotes was used to induce detectable inflammatory cytokine responses and pathology. For determining parasitemia and mortality, a dose below the 50% lethal dose of 75 parasites was used. During infection experiments, mice were kept under barrier conditions in the BSL 3 facility at the Research Centre Borstel in individually ventilated cages. All experiments were conducted according to the German animal protection laws and were approved by the Animal Research Ethics Board of the Ministry of Environment, Kiel, Germany. For *in vitro* experiments, *T. cruzi* parasites were cultured *in vitro* by weekly inoculation of semiconfluent LLC-MK2 cells with trypomastigotes drawn from supernatants of previously infected cells. *T. cruzi* culture trypomastigotes were harvested from LLC-MK2 cells, resuspended in Dulbecco’s Modified Eagle Medium (Biochrom), supplemented with 2 mM L-glutamine, 10% heat-inactivated fetal calf serum (Gibco), penicillin and streptomycin (100 U/ml and 100 μg/ml, respectively; Biochrom) and were directly used for *in vitro* experiments.

### Determination of parasitemia

Blood parasitemia was determined in 3 μl of tail vein blood that was lyzed in 27 μl NH_4_Cl (0.87% [wt/vol]) and viable parasites were counted in a hemocytometer as described previously^50^. Parasitemia was determined staring at day 7 post infection every two to four days.

### Quantification of tissue parasite burdens by quantitative real-time PCR

We used whole hearts and 50 mg of spleen, liver and intestine tissue for genomic DNA extraction and analyzed the tissue parasite burdens in these organs by quantitative real-time PCR. A 70 bp sequence of the 140/116-kDa antigen gene of *T. cruzi* (accession no. U15616) was amplified with the forward primer 5′-ACT CAT CGG GTT TGA AGC AT-3′, the reverse primer 5′-GCC AGG GTC TAG TAC TCT TTG CT-3′ and the internal probe 5′-CAG CAG GC-3′. A 107 bp stretch of the murine hypoxanthine-guanine phosphoribosyltransferase (*Hprt*) gene, used for quantification of host DNA, was amplified with the forward primer 5′-GTG GCC CTC TGT GTG CTC-3′, the reverse primer 5′-TCT ACA GTC ATA GGA ATG GAT CTA TCA-3′, and the internal probe 5′-ACC TGC TG-3′. Quantitative PCR was performed on a Light Cycler 480 (Roche Diagnostics). Data were analyzed employing the “Second Derivative Maximum Method” and “Standard Curve Method”.

### Histology

For histology, organs (one-half of spleens, whole hearts, the left liver lobes and 1 cm intestinal structures) were fixed in 4% formalin/PBS, set in paraffin blocks and sectioned (2 μm). Histopathological analyses were performed using standard protocols for H&E staining. For NOS2 staining, tissue sections were stained with a rabbit anti-mouse iNOS/NOS II antibody (Merck Millipore), a secondary biotin-labeled goat anti-rabbit antibody (Dianova), the Vectastain Elite ABC kit Standard (Vector Laboratories) and the DAB peroxidase substrate kit (Vector Laboratoties). For ARG1 staining, sections were stained using a goat anti-mouse arginase-1 antibody (Santa Cruz), a secondary biotin-labeled rabbit anti-goat antibody (Dianova) the Vectastain Elite ABC kit Standard (Vector Laboratories) and the DAB peroxidase substrate kit (Vector Laboratoties). NOS2- and ARG1-stained sections were counterstained with hematoxylin. To demonstrate collagen deposition, different organs were stained with azan trichrome and analyzed microscopically.

### AST, ALT and CK assay

Serum was prepared using the BD Microtainer SST Tubes (BD Pharmingen). AST, ALT and CK activity was determined in a volume of 32 μl serum using the Reflotron® System of Diagnosis (Roche Diagnostics).

### Quantification of mRNA expression of immune-related genes by quantitative real-time PCR

50 mg of organs (spleen, liver, heart, small intestine, colon and cecum) were homogenized in 4 M guanidinium-isothiocyanate buffer and total RNA was extracted by acid phenol extraction. cDNA was obtained using RevertAid H Minus M-MuLV reverse transcriptase (Fermentas) and random hexamer (Fermentas) as a primer. Quantitative PCR was performed on a Light Cycler® 480 Instrument (Roche Diagnostics). Data were analyzed employing the “Second Derivative Maximum Method” and “Standard Curve Method” using *Hprt* as a housekeeping gene to calculate the level of gene expression in relation to *Hprt*. The following primer and probe sets were employed: *Arg1*: sense 5′-CCT GGA ACT GAA AGG AAA G-3′, antisense 5′-TTG GCA GAT ATG CAG GGA GT-3′, probe 5′-TTC TTC TG-3′; *Hprt*: sense 5′-TCC TCC TCA GAC CGC TTT T-3′, antisense 5′-CCT GGT TCA TCA TCG CTA ATC-3′, probe 5′-AGT CCA G-3′; *Il22*: sense 5′-TTT CCT GAC CAA ACT CAG CA-3′, antisense 5′-TCT GGA TGT TCT GGT CGT CA-3′, probe 5′-CAG CTC CT-3′; *Lrg47*: sense 5′-AAG GCC ACT AAC ATC GAA TCA-3′, antisense 5′-TGC CTT ATC TCA CTT AAT ACT CCT CA-3′, probe 5′-CTC CTC TG-3′; *Nos2*: sense 5′-CTT TGC CAC GGA CGA GAC-3′, antisense 5′-TCA TTG TAC TCT GAG GGC TGA C-3′, probe 5′-AGG CAG AG-3′.

### Flow cytometry

Single cell suspensions of spleens were prepared at the indicated time points after infection and surface markers were stained with optimal concentrations of anti-CD4-V500, anti-CD8-V450, anti-CD62L-APC, anti-CD11c-FITC, anti-CD11b-PE (all from BD Bioscience), anti-CD44-FITC, anti-Gr1-APC (both from BioLegend), anti-CD90.2-APC-eFlour780 or anti-CD90.2-PE-Cy7 and anti-MHCII(I-A/I-E)-APC-eFlour780 (all from eBioscience). T_regs_ were stained by using the mouse regulatory T cell staining kit (eBioscience). For intracellular cytokine staining, 1 × 10^6^ cells were stimulated with plate-bound anti-CD3/anti-CD28 (each 5 μg/ml, BD Bioscience) for 4 h or with *T. cruzi* antigen for 12 h in the presence of GolgiPlug™ (BD Biosciences). Cell were stained with optimal concentrations of anti-CD4-V500, anti-CD8-V450 (both from BD Biosciences) and anti-CD90.2-APC-eFlour780 (eBioscience). Afterwards cells were fixed and permeabilized with Cytofix/Cytoperm™ (BD Biosciences). Intracellular accumulated cytokines were stained with anti-IFN-γ-APC (Biolegend), anti-IL-22-PE (R&D Systems) or the isotype control rat IgG2a-PE (R&D Systems). Data were acquired on a FACSCanto^TM^II (BD Bioscience) and analyzed with the FCS Express 4 Flow Cytometry software (DeNovo^TM^ Software). *T. cruzi* antigen was generated by repeated freezing and thawing of 1 × 10^8^ *T. cruzi* parasites in 1 ml PBS.

### Quantification of cytokine production

For the analysis of the IL-22 concentration in the sera of naïve and infected mice, serum was prepared using the BD Microtainer SST Tubes (BD Pharmingen) and the IL-22 concentration was determined by cytometric bead array according to the manufacturer’s instructions (Biolegend).

To analyze the cytokine secretion of spleen cells, single cell suspensions were prepared at the indicated time points after infection and 1 × 10^6^ cells were incubated in 300 μl Iscove’s Medium (Biochrom), supplemented with 2 mM L-glutamine, 10% heat-inactivated fetal calf serum (Biochrom), penicillin and streptomycin (100 U/ml and 100 μg/ml, respectively; Biochrom) for 24 h. The concentration of the indicated cytokines in the supernatant was determined by cytometric bead array according to the manufacturer’s instructions (BD Bioscience). Cytokine concentrations were analyzed using the FCS Filter and FCAP Array software (Soft Flow).

### *In vitro* killing of parasites by macrophages

To generate bone marrow-derived macrophages (BMDM), bone marrow cells were flushed from mouse femura of C57BL/6 mice and cultivated in L-929 conditioned medium as source for M-CSF activity for 11 days as described previously^7^. Cells were cultured in Chamber slides (Nalge Nunc international; 1 × 10^5^/well) overnight in Dulbecco’s Modified Eagle Medium (Biochrom), supplemented with 10% heat-inactivated fetal calf serum (Biochrom) and stimulated for 2 h with different cytokines in a total volume of 400 μl (IL-22: 100 ng/ml, R&D Systems, IFN-γ: 100 U/ml, BD Biosciences). Subsequently cells were infected with 50 μl *T. cruzi* culture trypomastigotes (2 × 10^5^/well) that were harvested from supernatants of infected LLC-MK2 cells. Extracellular parasites were removed at 2 h post infection and the cells were again incubated in the presence of the respective cytokines. 48 h post infection, cells were washed 3 times with PBS and stained with Hemacolor (Merck). The percentage of infected macrophages and the number of intracellular parasites per 200–400 cells were counted in triplicate cultures.

### Statistical analysis

Quantifiable data are expressed as means of individual determinations and standard errors. For the analysis of the parasitemia, the survival and the body weight change of mice infected with 50 parasites, 10 mice per group were used. For the analysis of immune responses and pathology of mice infected with 500 parasites, 5 mice per group were used. 2–5 uninfected mice per group were used as indicated in the legends. Statistical analysis was performed using the Mann-Whitney U test defining different error probabilities (*p ≤ 0.05; **p ≤ 0.01; ***p ≤ 0.001).

## Additional Information

**How to cite this article**: Erdmann, H. *et al.* During acute experimental infection with the reticulotropic *Trypanosoma cruzi* strain Tulahuen IL-22 is induced IL-23-dependently but is dispensable for protection. *Sci. Rep.* **6**, 32927; doi: 10.1038/srep32927 (2016).
